# Sanitary Convention at Ypsilanti, Michigan, July 1st, 1885

**Published:** 1885-08

**Authors:** 


### SANITARY CONVENTION AT YPSILANTI, MICHIGAN.

[Reported for the CHICAGO MEDICAL JOURNAL AND EXAMINER.]

Great interest was manifested in the Ypsilanti Sanitary Convention, which was held June 30th and July 1st, 1885, under the auspices of the Michigan State Board of Health. The physicians of Ypsilanti, the professors of the State Normal School, and a large number of other prominent citizens of that place were in constant attendance. About twenty physicians and health officers from other places were also in attendance.

C. L. Yost, Mayor of the city, opened the convention with a brief address of welcome, in which he said that "in times past we have been honored with the presence of other assemblages for consultation upon religious, political, medical or other interests; but this is the first time that disinterested philanthropists have come to us to discuss those great practical questions relating to the guardianship of public health." He thought that the adage, "an ounce of prevention is worth a pound of cure," was the inspiring motive that brought them together. Their city was more than usually lessed with conditions for good health; still they recognized the value of detailed instruction in sanitary subjects. Sanitary science is in its infancy, and all attempts to further it and bring intelligent attention to its teachings should be welcomed by thinking men everywhere.

Edwin Willits, President of the Michigan State Agricultural College, presided over the convention; and in his address dwelt upon the value of human life considered from an economic standpoint as worked out by eminent statisticians. A number of people who should produce $3,000,000 when in good health, could produce when not in good health only $2,000,000. Here is a loss of one-third, much of which could be saved by applying the simplest sanitary precautions. It is the duty of a community to keep these human earning-machines in good condition of health or earning-capacity, even from economical motives. A community has the legal right to do this. We want pure water and plenty of it, clean streets, cleansed sewers and drains, swamps drained, cesspools filled up; and we should not count the cost on our fingers. The crowning labor of our medical science is the warding off of disease. Given by heredity a sound constitution, pure air, pure water and nutritious food ought to guarantee to every human being, as he puts his foot on this earth, his three score years and ten, with which assurance he might plan the labors of a well-rounded life.

Rev. Dr. Woodruff, of Ypsilanti, read a paper on "The Moral Effect of Sanitation." He told of the importance the ancient Jewish people gave to sanitation; and in a forcible way pointed out its power in elevating man morally as well as socially and physically. He thought the present attention given to sanitary matters is not temporary. The reform has come to stay.

Prof. Austin George, of the State Normal School, spoke on "Sanitary Needs of School Buildings and Grounds." 1. School site should be on gravelly soil, a natural knoll, admitting thorough drainage; 2. Wells should not be nearer than 100 feet to the outhouses; 3. Basements should be high, with free circulation of air under ground floors. School rooms should contain 250 cubic feet of air space for each child. The light should come in over the left shoulder. The windows should go to the ceiling, and within three and one-half feet of the floor. The blackboard should be on the side of the room opposite the windows, and never have a glossy surface; 4. The simplest heating and ventilating apparatus is the hot-air furnace, and for small buildings the jacketed stove. The foul air outlets should be ample, and at the floor level. With any system, the rooms should be frequently flushed with fresh air through windows and doors. The temperature should be kept at 70° Fahr., not by teachers' sensations, but by thermometers; 5. Perfect desks and benches, not yet made; they should be adjustable, to suit different sizes, and be constructed with reference to the curves of the body. There should be foot-rests which could be raised or lowered until each pupil is made comfortable and able to take a healthful position. Many spinal complaints and pulmonary diseases are produced by improper desks; 6. Means for drying wet wraps should be provided; 7. Precautions against fire should be had, especially in the heating apparatus and the chimney; 8. "Inasmuch as the state educates the children, and compels their attendance in public buildings, the state should see that the children are not subjected to any preventable cause of disease."

This paper called out a long discussion, participated in by Dr. Avery, of Greenville, President of the State Board of Health; Prof. Vaughan, of Ann Arbor, member of the State Board of Health; Prof. McLouth of the State Agricultural College; Prof. Langley, of the Michigan University, and many others.

Dr. Bion Whelan, of Hillsdale, Mich., spoke on "Sanitation in Small Cities." The water supply should be guarded from contamination from cesspools and vaults. If we could not have sewers, the most feasible plan for disposing of garbage is to divide the city into districts and have it removed by licensed scavengers. Sanitary science should be taught in schools. The women should be taught sanitary needs, as much of sanitary work falls upon them. The nature of contagious diseases, and means for their restriction should be known by all.

Dr. J. H. Kellogg, of Battle Creek, member of the State Board of Health, treated of the "Disposal of Slops and Garbage," illustrating his remarks by use of the blackboard. He pictured in a graphic way the ordinary condition of back yards, as contrasted with front yards. He prefaced his subject proper with a brief description of bacteria and other low forms of microscopic life, and showed the relation of these to decay, no decay being possible without their intervention. He showed the importance and usefulness of certain sorts; the dangerous nature of others. He spoke of flies in relation to filth. They are a very strong indication of the presence of filth, and consequently of germs, and it is even believed that the germs of certain diseases may be spread by them. Whenever a house is full of flies, you may be certain that slops or garbage of some sort has been allowed to accumulate about the premises. He stated that the air about cesspools, foul drains and other filthy places is usually swarming with micro-organisms, and he believed that even if such germs were not the direct cause of certain diseases, the continual breathing of such air predisposes thereto. All sources of filth should be carefully and speedily removed from the habitation and its vicinity. Garbage can be reduced to a minimum by a little attention to domestic economy. Much food that becomes garbage through carelessness could be turned to good use if not neglected. Even after due care, however, some refuse will remain. So far as possible this should be burned. That is the best method of disposal. Where it is impracticable, a water-tight covered receptacle can be used for receiving such refuse, which should be removed frequently by a paid scavenger or otherwise. Slops should not be thrown upon the ground, into open drains or into cesspools in the yard. He had known of frequent instances in which wells and cisterns were contaminated from the seepage of foul privies, drains and cesspools. If slops must be poured on the ground, set off a portion of the back end of the garden, and distribute the slops on different parts of it on alternate days, giving in this way some opportunity for the soil to oxidize the organic matter. Slops containing excreta from contagious diseases should be thoroughly disinfected.

Chas. R. Whitman, of Ypsilanti, one of the regents of the Michigan University, read a paper on the "Limitations and Duties of Local Boards of Health." The powers and duties of local boards of health should be as follows:

1. To ascertain the causes of sickness and of death, and from time to time the prevailing diseases among all classes;

2. To prevent or mitigate diseases, especially zymotic or epidemic, endemic and contagious diseases;

3. To effect the periodical or special vaccination of the inhabitants;

4. To ascertain, prevent and remove or abate nuisances dangerous to public health;

5. To prevent the introduction of malignant, infectious or contagious diseases, and to isolate persons afflicted or believed to be afflicted with such diseases;

6. To establish, locate and manage hospitals for persons having contagious diseases;

7. To prevent the sale of any article for food or drink which is unwholesome;

8. To enforce these regulations by a sufficient penalty.

Mr. Whitman said that the radical defect in the law defining the powers and duties of local boards of health is the lack of a general law forbidding evils and imposing penalties. Such a law should be enacted, and it should be as sweeping and as readily enforced as is the criminal law. He thought that the subject of sanitation should become one of education in the schools, so that the future citizen, architect, and physician may be instructed in the principles of sanitation.

After a thorough discussion of this paper, participated in by Professor A. B. Palmer, of the University of Michigan, and many others, it was voted that Mr. Whitman be requested to draw a bill suitable for presentation to the legislature, framed to cover the question under discussion, and suited to correct existing defects in the present law. The bill is to be published in the proceedings of this convention.

Dr. A. F. Kinne, in a paper on "Sources of Malaria in Ypsilanti," took the ground that malaria is caused by a malarial germ native in certain soils and waters. Boards of health should require that the basements and sub-floor spaces be thoroughly ventilated; and no mill-dam owner who used water should be allowed to keep the pond much less than full.

Dr. O. W. Wight, Health Officer of Detroit, spoke on the "Prevention of Communicable Diseases." He showed clearly the contagious nature of those diseases; the great and unnecessary loss of human life resulting therefrom; and how they were best restricted, citing cases from his own long experience in Milwaukee and in Detroit. The main points in an outbreak of a contagious disease are:

1. On the part of the householder and physician—

(*a*). Speedy determination of the nature of the disease;

(*b*). Prompt notification to health-officer;

(*c*). Cheerful acquiescence in regulations of health-officer.

2. On the part of the health-department—

(*a*). Immediate separation of sick from the well, as only those required to nurse should be allowed in the room; the sick-room should be an upper chamber;

(*b*). Strict quarantine of the premises, and placarding of premises; if small-pox, all exposed should be vaccinated in both arms;

(*c*). Private funeral;

(*d*). Thorough disinfection, after death or recovery, by chlorine gas or fumes of burning sulphur, of bedding, clothing, premises, etc.

Dr. Wight thought that diphtheria and scarlet fever were sometimes spread by careless physicians,—criminal carelessness. During four years in Detroit he had witnessed a large growth of public sentiment in favor of public health-work.

Professor Victor C. Vaughan, of Ann Arbor, member of the State Board of Health, next spoke on "Water-Supply." Sources of water-supply are three—cisterns, surface, and subterranean. Cisterns should always be plastered outside and inside, with an excavation of at least two feet larger than the reservoir, so that a brick or stone wall may be built or plastered as above. Cistern water when boiled and filtered is the best for use. Professor Vaughan cited cases in Ann Arbor and Adrian in which drain-water percolated into cistern-water, causing malignant typhoid fever. In the discussion of surface-water, he said that we had in this country all the conditions for the development] and spread of the cholera-germ should it once reach our shores. Well-water is a suspicious water, because of the ease with which contamination can be carried dowu through the sand and gravel into the well. Subterranean water is not necessarily pure. By carefully selected illustrations he showed that contamination would be carried through the soil an almost limitless distance by the percolation of water, the water acting as the carrying agent.

Prof. Jas. H. Shepard, of Ypsilanti, read a paper on "The Present Condition of the Water Supply of Ypsilanti." The city has been settled 60 to 70 years; it has no sewers, and the privy system is in use. The drinking-water is obtained mostly from shallow wells. Ten careful analyses of well water had been made, and in eight of them chlorine and albuminoid ammonia were found largely in excess of what pure water should contain. The water of a drive-well, and that of the Huron river, which runs through the city, were comparatively pure.

A paper by Professor C. F. R. Bellows, of Ypsilanti, on "The Future Water Supply of Ypsilanti," was next read. It is an able paper, but is of a rather local interest, although the principle recommended may be quite generally adopted. The proposal is to have a general water-supply from one very large well outside the city proper, in such a locality that the watershed supplying the well shall not be liable to contamination by privies, etc.

The papers on water-supply called out a spirited discussion, led by Dr. Wight, of Detroit, and Professor Palmer, of the State University.

Dr. Ruth A. French, of Ypsilanti, read a paper on "Management of Earth-Closets." It is a practical way out of the "privy-nuisance" for towns too small to have sewers and a general water-supply. Earth-closets deodorize the excreta, destroy germs of contagious diseases, and are necessary to invalids.

Erwin F. Smith, of Lansing, spoke on the "Relations of Sewerage and Water-supply to the Death-rate in Cities." His general propositions were: —

1. Some method of sewage-disposal is a necessity of civilized life;

2. Dry-earth closets, *properly cared for*, will answer for isolated dwellings and small villages, but water-carriage is the only system adapted to large towns and cities;

3. The prevalence of typhoid and cholera is in an inverse ratio to the sewerage of a city;

4. The modern increase of diphtheria cannot be attributed to sewers;

5. The death-rate from all causes falls whenever a city is thoroughly sewered, and never attains its *ante-sewered* maximum;

6. Judged solely from the standpoint of pecuniary economy, —the lowest of all standards—sewerage and water-supply can be successfully defended against all opposition.

The statistics used in this paper are drawn from the highest authorities, American and foreign; are brought down to the close of the year 1884, and in many cases cover long periods—10 to 40 years. They show that typhoid fever has fallen off from one-half to nine-tenths in sewered cities since they adopted sewerage, and that such cities are practically secure from the ravages of cholera. *Per contra*, in non-sewered cities, the typhoid-fever and the cholera-mortality is as great to-day as it was thirty years ago.

Dr. H. F. Lyster, of Detroit, advocated the separate system of sewerage as best, and said it could be built in small cities for about $6.000 per mile. He spoke of the evil influence of soil-infection by vaults and privies, and urged sewerage as a sanitary necessity.

At the close of the convention, arrangements were made for the organization of a local sanitary association, the local committee of the convention being a committee to complete this organization.

